# Post-operative breast cancer patients diagnosed with skeletal metastasis without bone pain had fewer skeletal-related events and deaths than those with bone pain

**DOI:** 10.1186/1471-2407-10-423

**Published:** 2010-08-13

**Authors:** Mitsuru Koizumi, Masataka Yoshimoto, Fujio Kasumi, Takuji Iwase, Etsuro Ogata

**Affiliations:** 1 Molecular Imaging Center, National Institutes of Radiological Sciences, Anagawa 4-9-1, Inage-ku, Chiba, 263-8555 Japan; 2 Department of Nuclear Medicine, Cancer Institute Hospital, Tokyo 170-8455, Japan; 3 Department of Breast Surgery, Cancer Institute Hospital, Tokyo 170-8455, Japan; 4 Department of Internal Medicine, Cancer Institute Hospital, Tokyo 170-8455, Japan; 5 Breast Center, IUHW Mita Hospital, Tokyo, 108-8329, Japan; 6 Breast Center, Juntendo University, Tokyo 113-8431, Japan

## Abstract

**Background:**

Skeletal metastases are often accompanied by bone pain. To investigate the clinical meaning of bone pain associated with skeletal metastasis in breast cancer patients after surgery, we explored whether the presence of bone pain was due to skeletal-related events (SREs) or survival (cause specific death, CSD), retrospectively.

**Methods:**

Consecutive breast cancer patients undergoing surgery between 1988 and 1998 were examined for signs of skeletal metastasis until December 2006. Patients who were diagnosed as having skeletal metastasis were the subjects of this study. Bone scans were performed annually for 5, 7 or 10 years; they were also conducted if skeletal metastasis was suspected. Data concerning bone pain and tumor markers at the time of skeletal metastasis diagnosis, and data relating to various factors including tumors, lymph nodes and hormone receptors at the time of surgery, were investigated. The relationships between factors such as bone pain, SRE and CSD were analyzed using the Kaplan-Meier method and Cox's analysis.

**Results:**

Skeletal metastasis occurred in 668 patients but the pain status of two patients was unknown, therefore 666 patients were included in the study. At the time of skeletal metastasis diagnosis 270 patients complained of pain; however, 396 patients did not. Analysis of data using Cox's and Kaplan-Meier methods demonstrated that patients without pain had fewer SREs and better survival rates than those with pain. Hazard ratios regarding SRE (base = patients without pain) were 2.331 in univariate analysis and 2.243 in multivariate analysis. Hazard ratios regarding CSD (base = patients without pain) were 1.441 in univariate analysis and 1.535 in multivariate analysis. Similar results were obtained when analyses were carried out using the date of surgery as the starting point.

**Conclusion:**

Bone pain at diagnosis of skeletal metastasis was an indicator of increased SRE and CSD. However, these data did not support recommendations of follow-up bone surveys in breast cancer patients.

## Background

Skeletal metastasis is a common complication of breast cancer; more than 70% of patients who die from breast cancer exhibit osseous metastasis at autopsy [1]. However, the high incidence of metastasis at autopsy is not considered sufficient evidence to perform routine bone surveys on breast cancer patients [2]. The reason given is that there is no evidence that such surveys would improve patients' survival and quality of life [3-5].

There have been significant advancements in hormone therapy and chemotherapy for advanced breast cancer sufferers [6-8]. Furthermore, use of bisphosphonates to treat skeletal metastasis associated with breast cancer has been developed. Bisphosphonates reduce skeletal-related events (SREs) in patients with skeletal metastasis [9-13]. An analysis of the first appearance of skeletal metastasis in breast cancer patients revealed that patients with a solitary metastasis to bone (solitary bone lesion) had a better prognosis than those with multiple skeletal metastases (multiple bone lesions) [14]. These results suggest that early diagnosis of skeletal metastasis and treatment with bisphosphonates could improve a patient's quality of life by reducing the number of skeletal complications. There have been many studies investigating skeletal metastasis in breast cancer patients but most of these were conducted before the emergence of therapies such as bisphosphonates.

Pain is an important indicator of the presence of skeletal metastasis [15]. However, no clinical symptoms are evident in the early phase of skeletal metastasis. There are many prognostic factors related to survival, cause specific death (CSD). The relationship between these factors and SRE is not fully understood. Bone pain is a symptom produced by skeletal metastasis that could be related to SRE. The aim of the present retrospective study was to clarify the clinical meaning of bone pain at diagnosis of skeletal metastasis by investigating the relationship of pain to SRE and survival, and comparing this with other factors.

## Methods

### Patients

Breast cancer patients undergoing surgery at the Cancer Institute Hospital, Tokyo, Japan, between January 1988 and December 1998 formed the patient pool, and were followed up until December 2006. As the aim of this study was to clarify skeletal metastasis after surgery, patients with skeletal metastasis at the time of surgery were excluded from the pool (5429 eligible patients). Six hundred and sixty-eight patients developed skeletal metastasis during the duration of the study. The follow-up protocol after surgery was as follows: patients visited hospital and received a physical check every three months for the first two years, every six months thereafter until five years had elapsed, and then annually after that. Patients who received adjuvant therapy were followed up more frequently. Bone scanning was used to confirm the presence of skeletal metastasis; it was performed at the time of surgery for staging purposes, annually thereafter for five years, and then seven and 10 years after the date of surgery. Bone scanning was also performed if physicians suspected the presence of skeletal metastasis or wanted to exclude it as a possibility. In the event of positive or equivocal bone scans, other imaging techniques including X-ray, computed tomography and magnetic resonance imaging were used to confirm the diagnosis. The above procedures were conducted as routine clinical practice at the Cancer Institute Hospital, Tokyo, Japan.

### Pain and the values of tumor marker at the diagnosis of skeletal metastasis

The presence of bone pain at the time of diagnosis of skeletal metastasis was investigated using the card record, predominantly in the form of an inquiry card that was given to patients. In the inquiry card, patients were asked about the presence or absence of pain, and sites of pain. The grade of pain (visual-analog scale) was obtained from a limited number of patients. Therefore, the focus was on the presence or absence of pain. The values for tumor markers (carcinoembryonic antigen, CEA, and cancer antigen 15-3, CA 15-3) when osseous metastasis was diagnosed were classified as below, equal to or above the reference value. Reference values were 5.0 ng/ml for CEA and 30 ng/ml for CA 15-3.

### Factors at initial therapy

The following factors were used in a Cox proportional hazards mode: age at surgery (Age), menstruation state, breast tumor (T) with minor modification (T2 tumors were classed as small (2.1-3.0 cm) or large (3.1-5.0 cm)), clinical and pathological lymph node state (N and pN), histopathology, and estrogen receptor and progesterone receptor status. Tumors were classified as: in situ, non-invasive cancer; T0, no detectable tumor; T1, ≤ 2.0 cm; T2 small, 2.1-3.0 cm; T2 large, 3.1-5.0 cm; T3, ≥ 5.1 cm; T4, any size with direct extension to chest wall or skin according to the 2002 UICC-TNM classification [16]. Histological classification was performed according to the criteria of the Japanese Breast Cancer Society [17], which differs from the WHO classification. The Japanese system divides invasive ductal carcinoma not otherwise specified (NOS) according to the WHO classification into three categories based on morphology: papillotubular, solid-tubular and scirrhous carcinoma [17,18]. Lymph node status can be classified by two methods: N, clinical or preoperative classification; and pN, pathological classification. Pathological classification was used in this study as it is more accurate than clinical classification. The ER status of a tumor was classed as positive when the concentration was greater than 13 fmol/mg cytosol protein. The PgR status of a tumor was defined as positive when the concentration was greater than 10 fmol/mg cytosol protein.

### Analysis and statistical methods

Data were analyzed as follows. First, the percentages of patients with or without pain and with or without elevation of CEA or CA15-3 at the diagnosis of skeletal metastasis were calculated, and the time from surgery to the appearance of skeletal metastasis with or without pain was calculated and tested using the Mann-Whitney U test. Secondly, Kaplan-Meier's method with a log-rank test was used to compare SREs (pathological fracture, spinal cord paralysis, hypercalcemia, radiation therapy for osseous metastasis, orthopedic surgery for bone metastasis, and the use of morphine for pain) and survival (cause-specific death, CSD) in patients with and without pain at diagnosis of skeletal metastasis. Therefore, two types of events were used for the analysis: SRE and CSD. Deaths due to other causes were treated as censored. The starting point of the study was the date of diagnosis of skeletal metastasis. Thirdly, a Cox proportional hazards regression model was used to investigate whether the presence of pain was a significant factor using univariate and multivariate analysis. The proportionality of each hazard was confirmed by plotting a log-minus-log curve. Multivariate Cox proportional hazards regression analysis was conducted using a backward stepwise method (the statistical level of significance determined with the Wald test was set at P < 0.10). All analyses were performed using SAS version 8.2 (SAS Institute Inc., Cary NC). Finally, as a lead-time effect could influence the results, a similar analysis (second and third steps) using the initial surgery date as a starting point was also performed.

In addition, the number of bone metastatic lesions (solitary or multiple) at the time of diagnosis of skeletal metastasis was an important factor. The importance of this factor was reported [14] as solitary bone metastasis was a significant favorable prognostic factor compared with multiple bone lesions at initial presentation. Stratified sub-analysis was conducted on groups of individuals with a solitary bone lesion or multiple bone lesions at their initial presentation of skeletal metastasis.

This retrospective study was approved by the Institutional Review Board. Informed consent was not required as this was a retrospective study.

## Results

Of 668 patients who developed skeletal metastasis, the pain status was unknown in 2 patients, therefore 666 patients were included in this study. Of the total number of individuals, 396 (59.5%) did not complain of pain and 270 (40.5%) did. CEA values were available for 647 patients; 412 (63.7%) had a normal value and 235 (36.3%) were above the reference value. CA15-3 values were available for 649 patients; 391 (60.2%) had normal values and 258 (39.8%) were above the reference value. In 396 osseous metastasis patients without pain, 60 had elevated CEA without elevation of CA 15-3, 55 had elevated CA 15-3 without elevation of CEA, 80 presented with elevation of both CEA and CA 15-3, and in 201 patients both CEA and CA 15-3 were within normal limits. There were 187 (28.1%) patients who did not present with both bone pain and tumor marker elevation.

The patients' characteristics are presented in additional file 1. With the exception of CA15-3 and number of metastatic lesions (solitary or multiple), there was no significant difference between patients with and without pain regarding the factors noted at the start of therapy. The mean period from surgery to diagnosis of skeletal metastasis was 1262 days (median 1031 days) for patients without pain and 1268 days (median 921 days) for patients with pain. There was no statistical difference in the period from surgery to diagnosis between patients with or without pain (Mann-Whitney U test p = 0.785). The mean period from diagnosis of skeletal metastasis to development of SREs was 657 days (median 395 days) for patients without pain and 281 days (median 45 days) for patients with pain. There was a statistical difference in the period from diagnosis to development of SREs between patients with and without pain (Mann-Whitney U test p < 0.0001).

The numbers of patients with and without pain in each SRE are presented in additional file 2.

**Figure 1:**
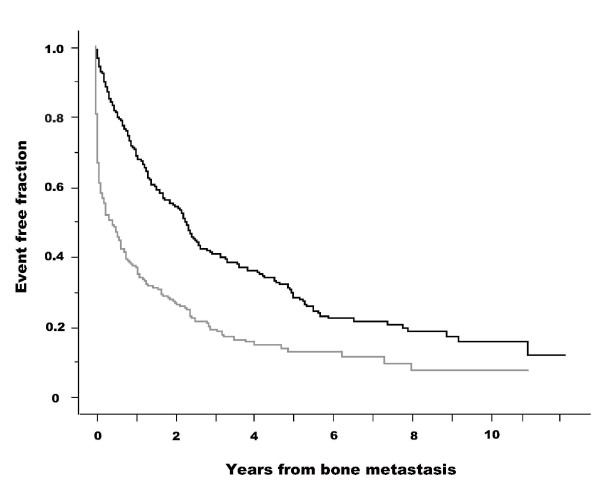
**Kaplan-Meier curves for skeletal-related events (SRE) in patients with and without pain**. Starting point was the date of diagnosis of skeletal metastasis. SRE fractions are shown for skeletal metastasis patients without pain (black line) and with pain (gray line). A log-rank test demonstrated a statistically significant difference between the two groups (p < 0.001). Starting point was the date of diagnosis of skeletal metastasis.

**Figure 2:**
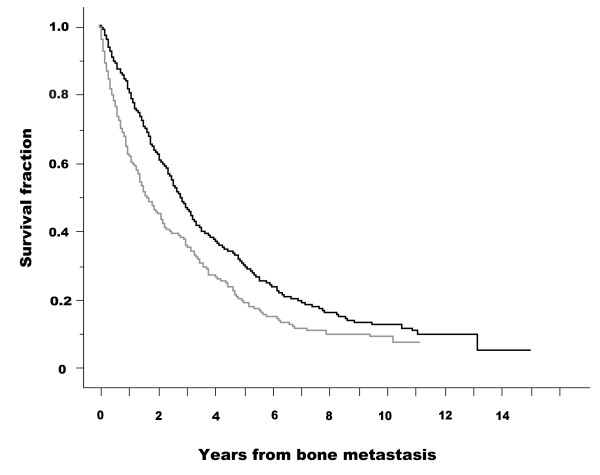
**Kaplan-Meier curves for survival (cause-specific death, CSD) in patients with and without pain**. Starting point was the date of diagnosis of skeletal metastasis. CSD fractions are presented for patients without pain (black line) and with pain (gray line). A log-rank test demonstrated a statistically significant difference between the two groups (p < 0.001). Starting point was the date of diagnosis of skeletal metastasis.

The Kaplan-Meier curves for SREs in patients with and without pain are presented in figure 1. The starting point was the date of diagnosis of skeletal metastasis. Patients without pain had fewer SREs than those with pain (log-rank test: *p *< 0.001). Figure 2 shows Kaplan-Meier curves for survival (CSD) in patients with and without pain. Patients without pain survived longer than those with pain (log-rank test: *p *〈0.001). The results of Cox's proportional hazard analysis regarding SRE are given in additional file 3. Presence or absence of pain was a significant factor in both univariate analysis (base: no pain, hazard ratio = 2.331, 95% CI 1.930-2.815) and multivariate analysis (hazard ratio = 2.243, 95% CI 1.815-2.773). The results of Cox's proportional hazard analysis regarding survival (CSD) are presented in additional file 4. Presence or absence of pain was a significant factor in both univariate analysis (base: no pain, hazard ratio = 1.441, 95% CI 1.211-1.715) and multivariate analysis (hazard ratio = 1.535, 95% CI 1.263-1.866).

**Figure 3:**
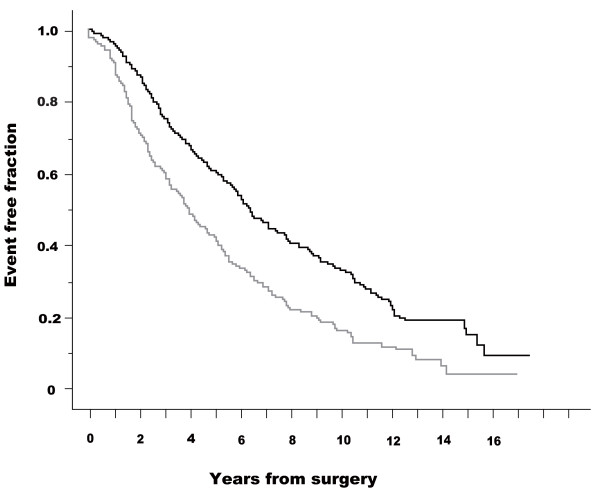
**Kaplan-Meier curves for skeletal-related events (SRE) in patients with and without pain**. Starting point was the date of initial surgery. SRE fractions are presented for skeletal metastasis patients without pain (black line) and with pain (gray line). A log-rank test demonstrated a statistically significant difference between the two groups (p < 0.001). Starting point was the date of initial surgery.

**Figure 4:**
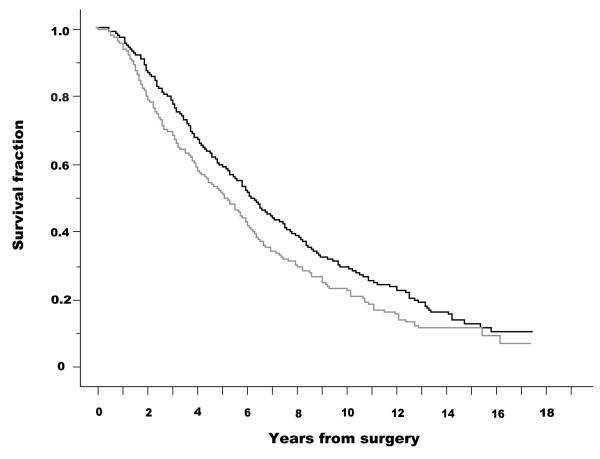
**Kaplan-Meier curves for survival (cause-specific death, CSD) in patients with and without pain**. Starting point was the date of initial surgery. CSD fractions are presented for patients without pain (black line) and with pain (gray line). A log-rank test demonstrated a statistically significant difference between the two groups (p = 0.07). Starting point was the date of initial surgery.

Figures 3 and 4 show Kaplan-Meier curves for SRE and survival (CSD) in patients without and with pain; the starting point was the date of initial surgery. These analyses (from the date of surgery) were carried out to neglect the lead-time effect of diagnosis. Patients without pain had fewer SREs than those with pain (log-rank test: *p *< 0.001). Patients without pain survived longer than those with pain (log-rank test: *p *= 0.007). Cox's proportional hazard analysis was performed for SRE and survival (CSD) from the date of surgery. The presence or absence of pain was a significant factor in both univariate analysis (base: no pain, hazard ratio = 1.691, 95% CI 1.402-2.040) and multivariate analysis (hazard ratio = 1.646, 95% CI, 1.343-2.018) with regard to SRE. Regarding survival (CSD), presence or absence of pain was a significant factor in both univariate analysis (base: no pain, hazard ratio = 1.268, 95% CI, 1.066-1.509) and multivariate analysis (hazard ratio = 1.365, 95% CI, 1.128-1.651).

**Figure 5:**
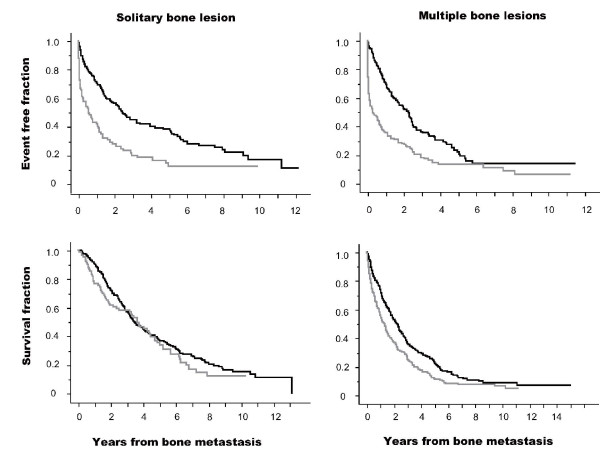
**Kaplan-Meier curves of SRE and CSD in patients with (gray line) and without pain (black line)**. Upper row figures indicate the analysis using SRE as an event. Lower row figures indicate the analysis using CSD as an event. Left line figures indicate the analysis in patients with a solitary bone lesion at the date of skeletal metastasis diagnosis, and right line figures indicate patients with multiple bone lesions. Starting point was the date of diagnosis of skeletal metastasis. Black lines indicate patients without pain and gray lines indicate patients with pain. A log-rank test in each patient group with solitary bone lesion and SRE event, solitary bone lesion and CSD event, multiple bone lesions and SRE event, and multiple bone lesions and CSD event were performed with χ2 values 27.4 (p < 0.0001), 0.8 (p = 0.36), 48.8 (p < 0.0001), and 15.8 (p < 0.0001), respectively.

The Kaplan-Meier curves for patients with or without pain are presented in figure 5. Patients with a solitary bone lesion and those with multiple bone lesions were analyzed separately. This analysis was performed using SRE and CSD as events and the date of diagnosis of bone metastasis as the starting point. In patients with a solitary bone lesion, individuals with pain showed a significantly higher incidence of SRE than those without pain but showed no significant differences in terms of CSD. In patients with multiple bone lesions, individuals with pain showed a significantly higher incidence of SRE and CSD than those with no pain but CSD incidence was less than that of SRE.

## Discussion

Bone pain is commonly associated with skeletal metastasis. However, in this study approximately 60% of patients did not complain of bone pain at diagnosis. Patients who developed skeletal metastasis without pain had fewer SREs and longer survival (CSD) than those with pain; these differences were statistically significant. The results were consistent irrespective of the analysis method used (Kaplan-Meier, log-rank test and Cox's proportional hazard model analysis). Cox's proportional hazards regression analysis using SRE and CSD as endpoints demonstrated that hazard ratios (univariate) of all significant factors were higher in value for CSD than for bone pain; this was also the case in multivariable analysis. Hazard ratio of bone pain was 2.331 for the SRE event, 1.441 for the CSD event (univariable analysis), 2.243 for SRE and 1.535 for CSD (multivariable). The presence of bone pain predicted SRE more accurately than CSD. Bone pain could be an important factor in predicting SRE, and other factors could be of more use in predicting CSD.

This research was a cohort study in a single institute that compared SRE and CSD of asymptomatic and symptomatic patients who had developed osseous metastasis. This type of comparison can be biased by lead time (early detection simply increases the period during which osseous metastasis is observed) and length time (patients with a long preclinical phase and therefore presumably less aggressive metastasis are likely to be detected by periodic bone survey). Differences in SREs and survival (CSD) could be due to the lead time bias. However, additional analysis taking the starting point as the date of surgery produced similar results. There was no difference in the chronological period of diagnosis of skeletal metastasis between patients with or without pain (data not shown), indicating that therapy after the diagnosis of skeletal metastasis did not differ between these groups of patients. There was no difference in the use of bisphosphonates for patients without pain and those with pain (data not shown). These findings indicate that fewer SREs and longer survival (CSD) were not due to the lead time bias. Length time bias could not be eliminated from this study even though the time from initial surgery to diagnosis of osseous metastasis was no different in patients with or without pain. Patients with painful bone metastases could have different disease biology from asymptomatic individuals (more aggressive recurrence, rapid growth, higher disease burden, more lytic lesions), which could account for their poor prognosis. The results presented herein do not predict that routine bone surveys will detect more asymptomatic patients and improve their outcome. However, the results do reveal that asymptomatic patients diagnosed with skeletal metastasis had lesser SRE and CSD than symptomatic patients. Kohno et al. reported that the SRE rate was higher among patients with pathological fractures before diagnosis than in patients with no prior fracture [12], and this could relate to the fact that the SRE rate was higher in patients with bone pain at the time of diagnosis of skeletal metastasis than in patients with no pain. Patients with skeletal metastasis but no bone pain (bone pain could be the result of micro-fractures at skeletal metastasis) should present with a lower SRE than patients with bone pain.

Drawbacks with this retrospective study include using a database of breast cancer patients in our institute, where data collection began approximately 30 years ago. The records lacked several important pieces of information including tumor grade and HER2/neu status, and it employed the local (Japanese) pathological classification system. A second database was utilized for data analysis of skeletal metastasis in breast cancer patients. This database lacked information regarding the type of skeletal metastasis (lytic, blastic, mixed type), and both databases lacked information regarding the pain condition of individuals. Patients' records (cards) were checked for the presence or absence of pain at diagnosis of skeletal metastasis but there was no detailed information about the pain condition of individuals.

Therapy for breast cancer has changed over time with the advancement of chemotherapeutic agents and their combination, hormones and molecular targeting agents (HER2/neu). The concept of standard therapy for skeletal metastasis has changed with the advent of bisphosphonates. In Japan, bisphosphonates were first approved for use in the treatment of skeletal metastasis at the end of 2004, and actual use began in 2005. Therefore, there were limited data regarding the use of bisphosphonates in this study and this prevented analysis of the impact of bisphosphonate treatment. The strategy of using radiotherapy for treating skeletal metastasis changed during the study period. The inclusion of radiotherapy treatment in the SRE classification could result in an overestimation of the incidence of SRE in this study. We have also performed another analysis in which first RT was not regarded as SRE and RT at least three months apart from first RT was counted as SRE. Although the distribution of kinds of SREs has been changed (additional file 5), the results of statistical analyses [Cox analysis (additional file 6) and Kaplan Meier analysis (data not shown)] were similar.

Patients were screened using periodic bone scans and more than 50% of patients (396/666, 59.5%) were diagnosed as having skeletal metastasis without pain. This is in contrast to other reports in which only a small percentage of patients were diagnosed with skeletal metastasis without pain. Front et al. reported that 21/66 (21%) patients did not have bone pain at diagnosis [19]. This difference could be due to the screening protocol used for skeletal metastasis, and advances in imaging modalities such as computed tomography and magnetic resonance imaging, which were used to confirm the presence or absence of skeletal metastasis.

Two randomized clinical trials (RCT) regarding breast cancer follow-up were published in 1994, although the studies were conducted almost 20 years ago. They concluded that regular physical examinations and annual mammography were as effective as more intensive approaches, based on laboratory and imaging tests in terms of timelessness of recurrence detection and overall survival and quality of life [3-5]. There have been no studies addressing this topic since then. However, the diagnosis and therapy of skeletal metastasis in breast cancer have advanced considerably since the two RCTs were conducted, and SRE was not used as an endpoint in those trials. Therefore, the results of the RCTs and the present study cannot be compared. Carrying out a RCT with SRE as an endpoint could be worthwhile.

Research into a new method of detecting skeletal metastasis using bone metabolic markers is underway [20]. Although the sensitivity of bone metabolic markers in detecting skeletal metastasis is currently insufficient, research into new markers is ongoing [20-22]. Another new technique used to detect metastasis, including skeletal metastasis, is positron emission tomography (PET). A number of publications conclude that PET is a good method for the detection of metastasis [23-25]. It is likely that bone surveys will be undertaken using methods other than bone scans, and that the clinical impact of bone metastasis will be clarified.

## Conclusions

Breast cancer patients who were diagnosed with skeletal metastasis without pain had fewer SREs than those with pain, and pain was a predictor of SRE. Other factors were predictors for CSD. Detection of skeletal metastasis in patients who do not have bone pain leads to a lower incidence of SREs. However, the data did not support a recommendation for bone surveys in the follow-up of breast cancer patients. These results could lead to a new randomized clinical trial to investigate whether early detection of skeletal metastasis has clinical significance.

## Competing interests

The authors declare that they have no competing interests.

## Authors' contributions

MK carried out planning of the study, data collection, analysis and interpretation, and writing the manuscript. MY, FK and TI performed patients' collection, management, and joined in discussions. All authors have read and approved the final manuscript. EO advised about the planning of the study and led the discussions, and he recently deceased.

## Pre-publication history

The pre-publication history for this paper can be accessed here:

http://www.biomedcentral.com/1471-2407/10/423/prepub

## Supplementary Material

Additional file 1 **Patient demographics and tumor characteristics**. Additional file 1 shows patients demographics and tumor characteristics. Click here for file

Additional file 2 **Skeletal related events and pain**. Additional file 2 shows skeletal related events with and without pain. Click here for file

Additional file 3 **Cox propotional hazard analysis as predicotors of SRE**. Additional file 3 shows the results of Cox propotional hazard analysis as predicotors of SRE. Click here for file

Additional file 4 **Cox propotional hazard analysis as predicotors of CSD**. Additional file 4 shows the results of Cox propotional hazard analysis as predicotors of CSD. Click here for file

Additional file 5 Revised skeletal related events* and pain. Additional file 5 shows revised skeletal related events and pain. Revised skeletal related events (SREs) were the events where the first RT was not counted as events. RT at least 3 months apart from the first RT was regarded as revised SRE. Click here for file

Additional file 6 **Cox propotional hazard analysis as predicotors of revised SRE**. Additional file 6 shows the results of Cox proportional hazard analysis as predictors of revised SRE. Revised skeletal related events (SRE) were the events where the first RT was not counted as events. RT at least 3 months apart from the first RT was regarded as revised SRE. Click here for file
